# An *in vitro* model to study immune activation, epithelial disruption and stromal remodelling in inflammatory bowel disease and fistulising Crohn’s disease

**DOI:** 10.3389/fimmu.2024.1357690

**Published:** 2024-02-12

**Authors:** Claire L. Mobbs, Nicole J. Darling, Stefan Przyborski

**Affiliations:** ^1^ Department of Biosciences, Durham University, Durham, United Kingdom; ^2^ Reprocell Europe Ltd, West of Scotland Science Park, Glasgow, United Kingdom

**Keywords:** inflammatory bowel disease, bioengineered, *in vitro*, disease modelling, barrier disruption, immune activation, extracellular matrix remodelling, fistulizing

## Abstract

At present, preclinical models of inflammatory bowel disease (IBD) are insufficient, limiting translation between research and new therapeutics. This is especially true for fistulising Crohn’s disease (CD), as the severe lack of relevant models hinders research progression. To address this, we present *in vitro* human IBD mucosal models that recapitulate multiple pathological hallmarks of IBD simultaneously in one model system - immune cell infiltration, stromal remodelling and epithelial disruption. Stimulation of models induces epithelial aberrations common in IBD tissue including altered morphology, microvilli abnormalities, claudin gene expression changes and increased permeability. Inflammatory biomarkers are also significantly increased including cytokines and chemokines integral to IBD pathogenesis. Evidence of extracellular matrix remodelling, including upregulated matrix-metalloproteinases and altered basement membrane components, suggests the models simulate pathological stromal remodelling events that closely resemble fistulising CD. Importantly, MMP-9 is the most abundant MMP and mimics the unique localisation observed in IBD tissue. The inflamed models were subsequently used to elucidate the involvement of TNF-α and IFN- γ in intestinal stromal remodelling, in which TNF-α but not IFN- γ induced MMP upregulation, specifically of MMP-3 and MMP-9. Collectively, our results demonstrate the potential of the IBD models for use in preclinical research in IBD, particularly for fistulising CD.

## 1 Introduction

Inflammatory bowel disease (IBD), including Crohn’s disease (CD) and ulcerative colitis (UC), is a chronic inflammatory disease of the gastrointestinal (GI) tract. Important pathophysiological hallmarks of IBD include leukocyte infiltration into gut mucosal tissue and barrier disruption resulting in a ‘leaky-gut’ phenotype. In addition to this, extracellular matrix (ECM) remodelling of the intestinal wall leads to tissue damage and severe complications (1, 2).

ECM remodelling is a hallmark of particular interest given distinct clinical disease subsets are characterised by different profiles of dysregulated ECM turnover. For example, structuring disease results from intestinal fibrosis, whereas fistulising disease is due to excessive ECM breakdown. These remodelling events are largely orchestrated by matrix metalloproteinases (MMPs), which are upregulated in IBD and correlate to the level of tissue destruction (3–5). Therefore, it is important that preclinical models encompass a stromal compartment to faithfully recapitulate disease complexity.

Standard clinical therapeutics for IBD are also inadequate, particularly in the prevention of tissue destruction, and are associated with severe adverse side effects (6). Newer therapies including biologic agents have demonstrated more success, yet 40% of patients remain non-responders. Moreover, approximately 90% of clinical trials for investigational new drugs (IND) fail, mainly attributable to the fact that most IND research is performed using ineffective animal models, time-limited *ex vivo* tissue and oversimplistic *in vitro* culture methods (7). Therefore, there is a major unmet requirement for improved preclinical *in vitro* models to facilitate the development of improved and safer therapies, particularly for addressing tissue remodelling complications.

While experimental animal models of IBD have been instrumental in the field of IBD, species differences restrict their biological relevance and translatability (8). Moreover, there is a severe lack of suitable animal models to study fistulising disease. At present, the only existing animal models include SAMP1/Yit mice, canine anal furunculosis (CAF), or TNBS rats with surgical creation of transsphincteric fistula (9). However, less than 6% of SAMP1/Yit mice develop fistulas, and the latter rat model is not relevant for the study of fistula formation (9, 10).

As an alternative, much knowledge has also been acquired through *in vitro* culture models. Simplistic Caco-2 cell cultures are used extensively throughout research, but monocultures of any cell type are insufficient in mimicking the multicellular intestinal environment in health or disease (11). To study the pathogenesis of IBD, more complex co-cultures and cell-culture substrates have thus been established to mimic disease characteristics such as epithelial disruption (12, 13). This has now been achieved in multiple co-culture systems through incorporation of an immune cell type, which is able to elicit an endogenous cytokine response (13–16). However, these models lack a stromal compartment and proper intestinal tissue microarchitecture.

Mucosal immunology was further revolutionised by the application of organoid technologies to disease research, which theoretically bridge the gap between *in vitro* culture and animal models (17). Recently, patient-derived organoids have been shown to exhibit translational and methylation differences compared to organoids generated from healthy tissue (18–20). Whilst these models hold huge potential as preclinical tools, they remain in their infancy. In addition, they are often inverted in structure and as such, do not represent the anatomy of the native tissue, and seldom include a fibroblast cell type (18, 21).

Incorporation of a mesenchymal component has only been reported in a limited number of *in vitro* IBD models (22, 23). Through stimulation of organoids with IL1a and TNF-α, one group were able to simulate fibrotic events such as altered ECM-composition and chemokine production (24). Jowett et al. further elucidated matrix-remodelling pathways through induced pluripotent stem cell (iPSC)-derived organoid-immune cell co-culture. Using this system, the authors successfully identified an immune-mesenchymal crosstalk mechanism involved in MMP expression, ECM-remodelling and subsequent tissue stiffening (25). However, while *in vitro* models to study pathological matrix remodelling in IBD are scarce, no *in vitro* model currently exists to study fistulising CD.

Moreover, no *in vitro* model has adequately recapitulated epithelial barrier dysfunction, immune cell activation and MMP-induced matrix-remodelling in one culture system that can be studied simultaneously. Whilst there are advantages to studying these pathways independently, there remains a requirement for a model encompassing the numerous pathological hallmarks simultaneously to enable more effective drug-testing and research progression.

In this study, we present an *in vitro* IBD mucosal tissue equivalent that exhibits these disease hallmarks. These relatively simple and cost-effective models have the potential to serve as a fundamental technological tool in IBD research and IND development, supplementing or replacing existing outdated pre-clinical drug screening methods. Given the sources of the cells used to construct these models, the resulting tissue constructs were found not to be limited by the availability of sufficient cell numbers, cell population purity, or insufficient differentiation, and were always robust and reproducible. Furthermore, the model presents opportunity to be used to study pathological matrix remodelling typical of fistulising CD.

## 2 Materials and methods

### 2.1 Cell maintenance and monocyte differentiation

Caco-2 cells (ECACC, Porton Down, UK) and neonatal human dermal fibroblasts (HDFn, Thermo Fisher Scientific, Loughborough, UK) were maintained in complete DMEM supplemented with 10% foetal bovine serum (FBS), 1% penicillin/streptomycin, 2 mM L-glutamine, and 1% non-essential amino acids (NEAA – all Thermo Fisher Scientific). Cultures were maintained at 37°C, 5% CO_2_ in a humidified incubator and passaged following manufacturer guidelines using 0.25% Trypsin EDTA (Thermo Fisher Scientific UK). THP1-Blue™ NF-κB Cells (Invivogen, UK), hereafter named THP-1-NF-κB, were maintained in Roswell Park Memorial Institute (RPMI) 1640 media (Thermo Fisher Scientific) supplemented with 10% heat-inactivated FBS, 1% penicillin/streptomycin, 2 mM L-glutamine, 1% NEAA in a humidified incubator at 37°C, 5% CO_2_. THP-1-NF-κB were derived from THP-1 monocyte cell line by stable integration of an NF-κB inducible secreted alkaline phosphatase (SEAP) reporter construct that allows monitoring of NF-κB activation by determining SEAP activity. Maintenance medium was supplemented with 10 µg/mL blasticidin (Invivogen) every alternate passage to maintain selection pressure. For THP-1-NF-κB cell differentiation, cells were seeded into T175 flasks (Sarstedt, Nümbrecht, Germany) at 0.8x10^6^ cells/mL and incubated for 48 hours in RPMI-1640 supplemented with 100 ng/ml phorbol 12-myristate 13-acetate (PMA, Thermo Fisher Scientific). Differentiated macrophages were detached using 1X TrypLE™ Express Enzyme (Sigma Aldrich).

### 2.2 Generation of IBD mucosal constructs

*In vitro* IBD mucosal constructs were generated using Alvetex^®^ Scaffold inserts (Reprocell Europe Ltd, UK). Scaffolds were prepared following manufacturer instructions before cell seeding. IBD mucosal constructs were adapted from our previously published intestinal models by incorporation of a viable immune component (26). Firstly, a fibroblast tissue layer was generated within the Alvetex^®^ Scaffold. For this, 0.5x10^6^ HDFn cells were seeded onto inserts and cultured for 12 days in complete DMEM supplemented with 5 ng/mL TGF-β1 (Peprotech, London, UK) and 100 μg/mL ascorbic acid (Sigma Aldrich, UK); medium was replaced every 3-4 days. On day 12, immune-competent lamina propria compartments were established through addition of 0.5x10^6^ pre-differentiated THP-1-NF-κB macrophages onto the fibroblast tissue equivalent and cultured in complete DMEM for a further 2 days. Caco-2 cells were then added to the 3D cultures on day 14 at a density of 0.4x10^6^ cells/insert and were cultured in complete DMEM for an additional 21 days, during which the medium was replaced every 3-4 days.

### 2.3 Inflammatory activation of IBD mucosal constructs

To stimulate an inflammatory microenvironment, IBD mucosal constructs were stimulated with 1 μg/mL lipopolysaccharide (LPS), derived from *Escherichia coli (E. coli)*, serotype O55:B5 (Sigma Aldrich) in complete DMEM for 24-96 hours. LPS supplemented DMEM was changed every 24 hours. For cytokine stimulation of models, 10 ng/mL recombinant human TNF-α (R&D systems, Minneapolis, USA), or 10 IU/mL recombinant human IFN- γ (Peprotech) was added to complete DMEM every 24 hours for 96 hours.

### 2.4 Quantification of nuclear NF-kB induced SEAP

To determine NF- κB activity in THP-1-NF-κB macrophages within IBD models, QUANTI-Blue™ (QB - Invivogen) assays were performed. Upon stimulation with LPS, NF-κB activation results in secreted embryonic alkaline phosphatase (SEAP) release into culture medium. QB solution was prepared as per manufacturer’s instructions and 180 μL was added per well of a flat-bottom 96-well plate (Sarstedt). 20 μL of culture medium was taken from models post-stimulation and added to each well of the 96-well plate and incubated at 37°C for 10 minutes. SEAP levels were subsequently determined using a spectrophotometer at 655 nm.

### 2.5 Secreted inflammatory biomarker quantification

Post-stimulation cell culture supernatants were collected and analysed in triplicate via bead-based multiplex technology (Eve Technology, Calgary, AB). Inflammatory cytokines and chemokines were quantified by Human Cytokine/Chemokine 48-Plex Discovery Assay^®^ array, and MMPs/TIMPs were quantified by Human MMP and TIMP Discovery Assay^®^ array.

### 2.6 Assessment of barrier integrity and paracellular permeability

Epithelial integrity was determined by measuring transepithelial electrical resistance (TEER) with an Epithelial Volt Ohm Meter (EVOM2) equipped with STX2 chopstick electrodes. Final TEER values, represented as Ω*cm^2^, were determined by subtraction of the blank insert TEER value and multiplying by cell-surface area of the Alvetex^®^ scaffold (Equation 1):


$$\text{TEER }\left(Ω.{\text{cm}}^{2}\right)\;=\;\left(Ω\text{ of model }–\;Ω\text{ of blank insert}\right)\;*\text{ insert surface area }\left({\text{cm}}^{2}\right)$$

TEER values are represented as percentage normalised to unstimulated control values.

Paracellular permeability assays were performed to assess the permeation of FITC-labelled dextran-20 (FD20) across the epithelial layer of the IBD models in the apical-basal direction. Transport assays were conducted in Hanks Buffered Saline Solution (HBSS) (Sigma Aldrich). Cell culture medium was removed from models before washing twice in PBS. Models were then placed in 12-well plates and supplemented with 1.5 mL HBSS basally and 0.4 mL apically with HBSS containing FD20. Cultures were incubated for 1 hour at 37°C. Samples were then taken from the basal compartment and values determined using a plate reader. Apparent permeability coefficient was calculated as follows:


Papp (cm/s) = (VR∗(dCR/dt))/(A*CD0)

VR is the volume of the receiver compartment; dCR/dt is the change in concentration of the analyte over time; A is the transport interface surface area and CD0 is the analyte concentration of the donor compartment at the start of the experiment.

### 2.7 Paraffin embedding and histological analysis

IBD mucosal constructs were PBS washed and fixed in 4% paraformaldehyde (Thermofisher Scientific) for 2 hours. Samples were dehydrated in a graded ethanol series and incubated in Histoclear II (National Diagnostics, United States), before they were embedded in paraffin wax (Thermofisher scientific) using embedding moulds (CellPath, Newton, UK). 5 μm transverse sections were mounted for haematoxylin & eosin (H&E) staining. For this, slides were deparaffinised in Histoclear II prior to gradual rehydration in ethanol. Slides were incubated in Mayer’s haematoxylin (Sigma-Aldrich) for 5 minutes, washed in deionised water then incubated in alkaline ethanol to blue the nuclei. Slides were then dehydrated to 95% ethanol, counterstained with eosin (Sigma Aldrich) before being dehydrated with dry ethanol and mounted using Omni-mount (National diagnostics). All histological samples were imaged using a Leica microscope.

### 2.8 Immunostaining

For immunofluorescence analysis, paraffin-embedded sections were first deparaffinised in Histoclear II and then rehydrated through an ethanol series. Heat-mediated antigen retrieval was achieved by incubation in citrate buffer (Sigma Aldrich) for 20 minutes. Samples were then permeabilised in 0.4% Triton X-100 (Sigma Aldrich) and blocked in 20% normal calf serum (NCS - Thermofisher Scientific) for 1 hour. Primary antibodies were diluted in blocking buffer and applied overnight at 4°C (**Supplementary Table 1**). Following this, samples were washed 3 times in PBS before incubation in Alexa Fluor-conjugated secondary antibodies and Hoechst nuclei stain (Thermofisher Scientific) diluted 1:1000 in blocking buffer before mounting in Vectashield (Vector Labs, Peterborough, UK). Images were acquired using Zeiss 880 confocal microscope.

### 2.9 Quantification of proliferation and apoptosis

To calculate proliferation, paraffin-embedded sections were immunolabelled with Ki67 and counterstained with Hoechst (1:1000). To detect apoptotic cells, terminal deoxynucleotidyl transferase dUTP nick end labelling (TUNEL assays (Promega) were performed on paraffin-embedded sections, and counterstained with Hoechst (1:10,000). 5 non-overlapping images were acquired per model to calculate mean percentage per model. Labelled sections were imaged using a Zeiss 880 confocal microscope. Ki67/TUNEL^+^ cells were counted and calculated as a percentage of total epithelial cells per image.

### 2.10 Transmission electron microscopy

For TEM analysis, samples were fixed in Karnovsky’s Fixative consisting of 8% PFA, 25% glutaraldehyde (Agar Scientific, Stansted, UK), and 0.2 M cacodylate buffer (Agar Scientific) for 1 hour before washing in 0.1 M cacodylate buffer. Samples were further fixed in 1% osmium tetroxide (Agar Scientific) for 1 hour, washed in 0.1 M cacodylate buffer before being dehydrated through an ethanol series before embedding in Epon resin (Agar Scientific) containing dodecenylsuccinic anhydride (DDSA), methyl nadic anhydride (MNA) and benzyldimethylamine (BDMA). Ultra-thin (70 nm) sections were obtained using a diamond knife (Agar Scientific) and loaded onto formvar-coated slot grids (Agar Scientific). Grids were then stained with uranyl acetate (Agar Scientific) and lead citrate (Sigma Aldrich) prior to imaging using a Hitachi H7600 electron microscope.

### 2.11 Western blotting

IBD mucosal constructs were washed in PBS before being lysed in ice-cold Mammalian Protein Extraction Reagent (MPER – Thermofisher Scientific) containing 1% HALT™ protease and phosphatase inhibitor cocktail (Thermofisher Scientific). Lysates were homogenised and pelleted for 20 minutes at 12,000 rpm before subsequent harvesting of protein supernatant. To quantify the protein content of the supernatant, Bradford assays (Bio-Rad, Oxford, UK) were performed following manufacturer instructions. 30 μg of sample protein, diluted 4:1 with 90% Laemmli buffer (Bio-Rad): 10% 2-meraptoethanol (Sigma Aldrich) was heated to 95°C for 5 minutes before loading into SDS-PAGE gels, electrophoresed and transferred onto a 0.45 μM nitrocellulose membrane (GE Healthcare Life Sciences, Buckinghamshire, UK) via wet-transfer. Membranes were blocked in 5% milk in Tris-buffered saline (TBS-T) before incubation in primary antibody diluted in 5% milk-TBS-T overnight at 4°C (**Supplementary Table 1**). Membranes were washed in TBS-T before incubation in appropriate horseradish peroxidase (HRP)-conjugated secondary antibodies (Sigma Aldrich) diluted 1:10,000 in 5% milk-TBS-T for 1 hour at room temperature. Clarity Enhanced Chemiluminescence (ECL, Bio-Rad) was used to detect antibody binding.

### 2.12 mRNA isolation and RT-qPCR

RNA was extracted from IBD mucosal constructs using Reliaprep™ RNA Tissue Miniprep System (Promega, Southampton, UK) following manufacturer instructions. 1 μg of complimentary-DNA was generated using High Capacity cDNA Reverse Transcription (RT) kit (Applied Biosystems, Thermofisher Scientific). RT-qPCR detection was performed based on SsoAdvanced SYBR green (Bio-Rad) using a CFX Connect Real-Time PCR System. Assays were run in triplicate. Quantification was performed using ΔΔCt method to obtain fold change of the desired gene in stimulated samples relative to unstimulated controls, and signal was normalised to housekeeping gene *GAPDH*. KiCqStart Primers (Sigma Aldrich) were used and are listed in **Supplementary Table 2**.

### 2.13 Human tissue acquisition and use

Fixed healthy and diseased human intestinal tissues were obtained by Reprocell (Glasgow, United Kingdom) following appropriate ethical and consent protocols, ensuring compliance with local laws and regulations. Tissues were received at Durham University under a formal MTA agreement and were processed following relevant UK HTA rules and guidelines at the time of publication.

### 2.14 Statistical analysis

Statistical analysis was performed on data from 3 independent biological experimental repeats unless otherwise stated in the figure legend. All analyses were carried out using Graphpad Prism 9 and data are expressed as mean ± SEM. Normality of data was tested using Shapiro-Wilk test. All data containing multiple groups were analysed using a one-way ANOVA with Tukey’s *post-hoc* multiple comparisons test. *P < 0.05, **P < 0.01, ***P < 0.001, ****P < 0.0001 were accepted as significant.

## 3 Results

### 3.1 Development and characterisation of three-dimensional *in vitro* tissue constructs of the IBD mucosa

**Figure 1:**
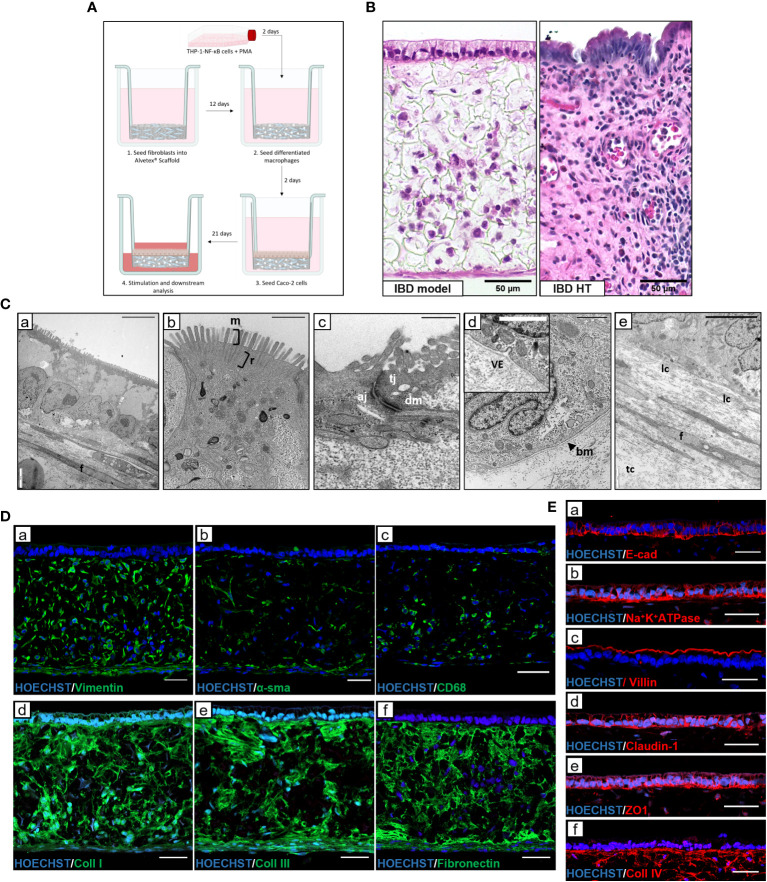
Development and characterisation of *in vitro* IBD mucosal constructs. **(A)** Workflow schematic for model set-up using Alvetex^®^ Scaffold. **(B)** H&E image of unstimulated IBD model and IBD human tissue (HT) for comparison. Scale bars 50 μm. **(C)** TEM micrographs of epithelial polarisation **(C**,a**)**, and microvilli (m) brush border with clear rootlet (r) visibility **(C**,b**)**, apical-junctional complexes consisting of tight junction (tj), adherens junction (aj) and desmosome (dm) **(C**,c**)**, basement membrane (bm) formation with vesicle exocytosis (ve) **(C**,d**)**, and the lamina propria composition containing fibroblasts (f) and longitudinal collagen and transverse collagen (lc and tc) **(C**,e**)**. Scale bars: 10 μm **(C**,a**)**, 1 μm (**C**,b + **C**,d), 0.5 μm **(C**,c**)** and 5 μm **(C**,e**)**. **(D)** Representative IF images of fibroblast markers (**D**,a + **D**,b), pan-macrophage marker CD68 **(D**,c**)** and ECM protein expression (**D**,d, **D**,e + **D**,f) within Alvetex^®^ lamina propria-like compartment. Scale bars 50 μm. **(E)** IF staining of epithelial and polarisation markers in IBD model epithelial tissue. Scale bars 50 μm.

To effectively model IBD *in vitro*, a physiologically relevant microenvironment must be recapitulated. To achieve this, we used Alvetex^®^ scaffold technology – an inert, porous polystyrene scaffold - to establish three-dimensional (3D) co-cultures representative of the IBD mucosa. A lamina propria-like compartment was created through co-culture of fibroblasts and immune cells within and on the surface of the scaffold, supporting the formation of an overlying epithelium, as outlined in **Figure 1A**. Macrophage-like cells were included as they are a predominant cell type involved in both innate and adaptive mucosal immunology. Histological analysis of IBD mucosal constructs revealed a confluent 3D fibroblast layer populated with macrophages, subjacent to a columnar epithelium (**Figure 1B**). Electron micrographs confirmed polarisation of the epithelium containing basal nuclei (**Figure 1C**). A confluent microvilli brush-border covered the epithelium with visible rootlets projecting below the cell membrane. Moreover, apical-junction complexes consisting of tight junctions, adherens junctions and desmosomes formed between adjacent epithelial cells. Electron dense layers at the epithelial-stromal show clear tri-lamina basement membrane formation and vesicular crosstalk between the tissue compartments. Within the lamina propria, fibroblasts produce a dense ECM comprising longitudinal (lc) and transverse collagen (tc) fibres.

Immunofluorescence characterisation of the lamina propria compartment (**Figure 1D**) revealed a heterogeneous population of vimentin^+^ and αSMA^+^ fibroblasts, reflective of a fibrotic IBD myofibroblast-like phenotype. CD68^+^ macrophages also populated the protein-rich ECM compartment. Abundant endogenous ECM was secreted by resident fibroblasts, creating an instructive tissue-like microenvironment in which cells reside and migrate. This ECM network contained essential intestinal ECM proteins including collagen-I, -III, IV and fibronectin. Epithelial proteins, including differentiation marker villin, junctional proteins including E-cadherin, claudin-1, and ZO1 as well as functional polarisation marker Na^+^K^+^ATPase (**Figure 1E**) demonstrate the presence of essential barrier components. In addition, localised collagen-IV staining indicated the presence of a basement membrane (BM).

### 3.2 Induction of an inflammatory response in IBD mucosal models recapitulates pathological inflammation and damage to tissue microarchitecture

**Figure 2:**
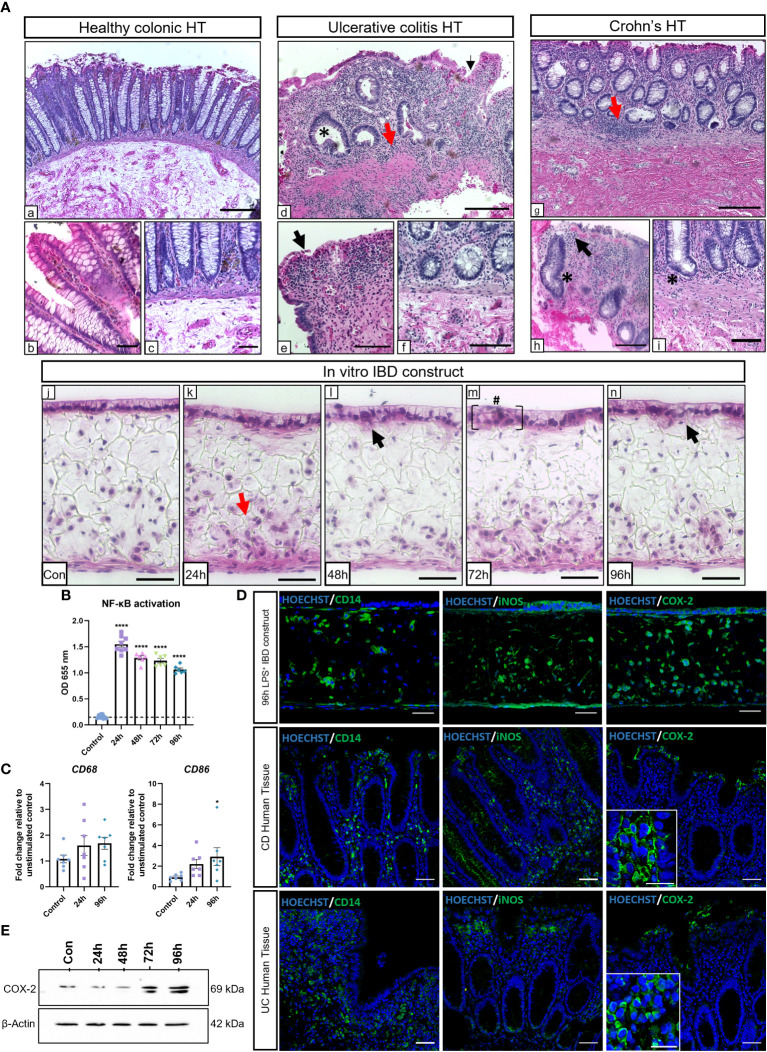
LPS stimulation of IBD mucosal models induces histological damage and inflammatory marker upregulation. IBD mucosal constructs were repeatedly stimulated with LPS every 24h for 0-96h. **(A)** H&E of healthy colonic **(A**,a-c**)**, ulcerative colitis colonic **(A**,d-f**)** and Crohn’s disease colonic **(A**,g-i**)** human tissue, and 0-96h stimulated IBD mucosal constructs **(A**,j-n**)**. Representative images show histological disease characteristics including basal lymphocytosis (red arrows), altered epithelial architecture (black arrows), crypt atrophy and abscesses (*), and epithelial hyperplasia (#). Scale bars: A.a,d+g: 200 μm, A,b,c,e,f, h+i: 100 μm, and A,j-n: 50 μm. **(B)** LPS stimulation of IBD constructs increases NF-κB activation, n*=*6-9 from 3 independent experiments. ****p< 0.0001 compared to control. **(C)** Relative mRNA expression of *CD68* and *CD86* in control and stimulated IBD mucosal models. N=7. * p< 0.05. **(D)** IF images of inflammatory marker localisation in 96h LPS stimulated IBD constructs compared to CD and UC. Scale bars 50 μm. **(E)** Western blot depicting COX-2 upregulation within stimulated models. β-Actin used as loading control.

To evaluate the potential of the IBD mucosal constructs to be used as an *in vitro* tool for disease research, we aimed to induce an inflammatory phenotype that mimicked hallmarks of disease. Models were stimulated daily with LPS for 24-96h, and histological findings were compared to healthy and diseased human tissue (**Figure 2A**). Common histopathological changes were observed between healthy and diseased human tissue. Similarly, we observed evidence of IBD histopathology in inflamed IBD models including time-dependent epithelial disruption and loss of epithelial polarisation and organisation. Moreover, epithelial breaches became apparent following 48-hours of stimulation (black arrows) which resulted in areas of cellular invasion into the lamina propria compartment similar to the epithelial distortion observed at atrophic crypts in the diseased tissue. Areas of epithelial pseudostratification were also identified following stimulation, matching observations in UC tissue (27). Incorporation of macrophages recapitulated the cellular abnormality of diseased tissue following application of a proinflammatory stimulus.

The NF-κB transcription pathway is largely upregulated in IBD and is a major regulatory component responsible for many inflammatory responses (28). NF-κB activation was significantly higher in macrophages within stimulated IBD models than unstimulated controls (**Figure 2B**). Increased expression of *CD68* and *CD86* were indicative of M1 macrophage polarisation in stimulated models (**Figure 2C**). Inflammatory markers associated with IBD were then investigated and compared to IBD tissue (**Figure 2D**). Comparable CD14^+^ macrophage staining was observed in diseased tissue and stimulated models. Inflamed IBD models also exhibited similar expression of nitric-oxide synthase (iNOS) to diseased tissue. iNOS expression was localised to the epithelial layer and infiltrating leukocytes in both CD, UC and inflamed IBD mucosal models. iNOS^+^ fibroblasts were also evident within IBD mucosal models. Cyclooxygenase-2 (COX-2) is a pro-inflammatory enzyme upregulated in IBD tissue (29). The expression of COX-2 was particularly evident in epithelial and immune cells in IBD tissue and inflamed IBD models. Western blot analysis revealed a time-dependent increase in COX-2 in stimulated models (**Figure 2E**), suggesting induction of chronic inflammation.

### 3.3 Inflammatory cytokines are secreted from inflamed IBD mucosal constructs

**Figure 3:**
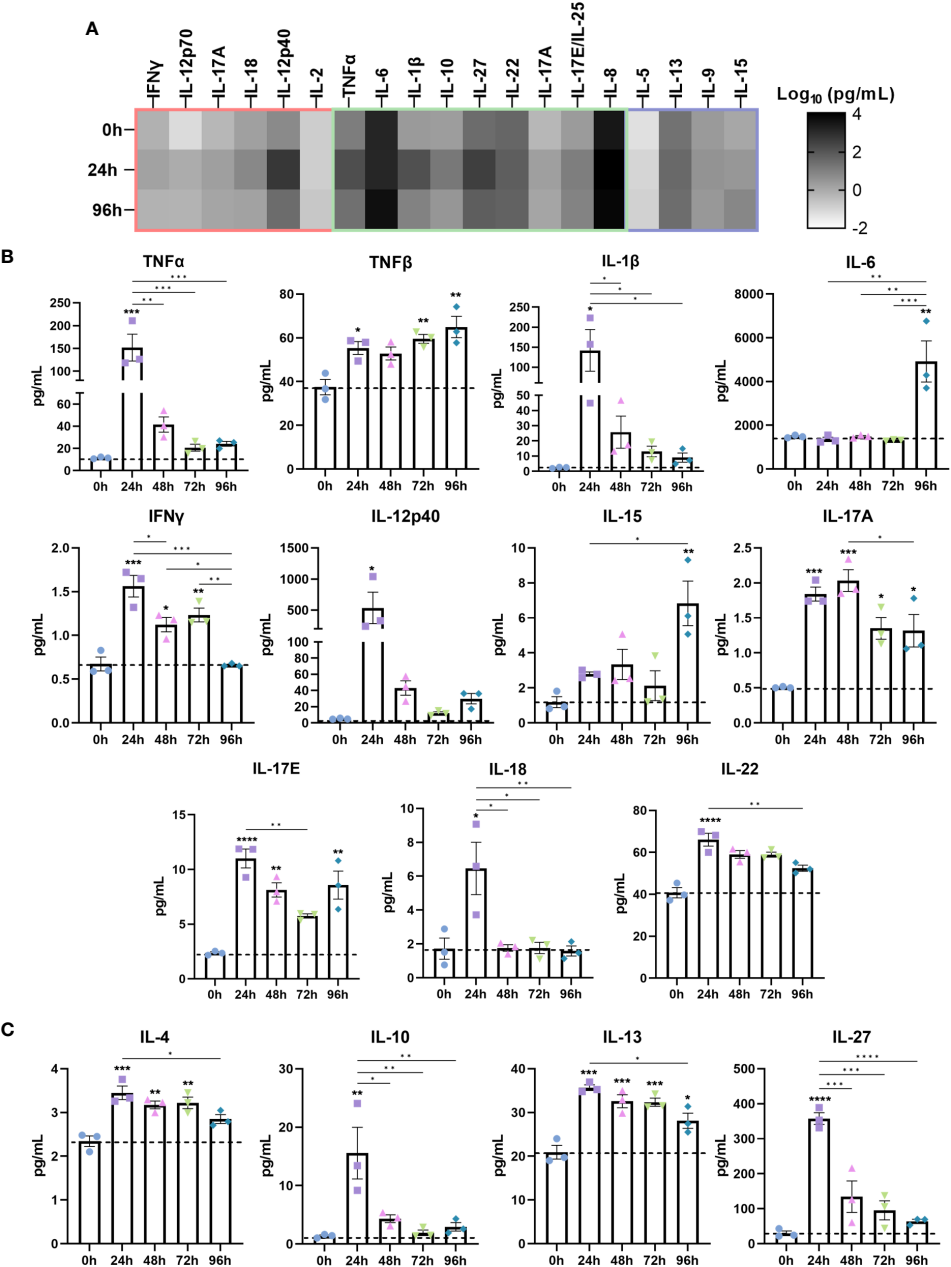
Stimulated IBD mucosal constructs elicit pathophysiological cytokine responses observed in CD and UC. Cytokine content of cell-culture media of each model was quantified by Luminex multiplex platform. Data are presented as mean ± SEM from 3 independent experiments: *p< 0.05, **p< 0.01, ***p< 0.001, ****p< 0.0001. **(A)** Heatmap of cytokines categorised according to the cytokine signature of CD (red), UC (purple) or those common to both (green). The colour intensity indicates the Log_10_ of the mean pg/mL for each cytokine. **(B)** Level of pro-inflammatory cytokines 0-96h following LPS administration to IBD mucosal models. **(C)** Level of anti-inflammatory cytokines following 0-96h LPS stimulation.

Next, we investigated the cytokine secretome of IBD mucosal constructs following LPS stimulation (**Figure 3**). When grouped according to inflammatory signature into CD-typical TH1/TH17, UC-atypical TH2 (**Figure 3B**), and cytokines common to both pathologies (**Figure 3A**), it was clear that the stimulated secretome exhibited features of both UC and CD, though IL-5 expression was negligible throughout.

Significantly upregulated cytokines included pro-inflammatory TNF-α, TNF-β, IL-1β, IL-6, IFN-γ, IL-12p40, IL-15, IL-17, IL-18 and IL-22 (**Figure 3A**). Anti-inflammatory cytokines including IL-4, IL-10, IL-13 and IL-27 were also upregulated (**Figure 3B**). Differential trends in the temporal regulation of cytokines were evident. Some cytokines were most significantly upregulated following 24 hours of stimulation and decreased in a time-dependent manner. In contrast, TNF-β, IL-6 and IL-15 exhibited a time-dependent increase. Cytokines associated with epithelial dysfunction, including TNF-α, IL-1β, and IL-6 were significantly heightened in inflamed models (30). Surprisingly, IL-6 levels exhibited a profound increase following 96 hours stimulation, suggesting initiation of chronic inflammation as IL-6 functions in orchestrating the chronic inflammatory response (31).

### 3.4 Inflammatory chemokines are secreted from inflamed IBD mucosal constructs

**Figure 4:**
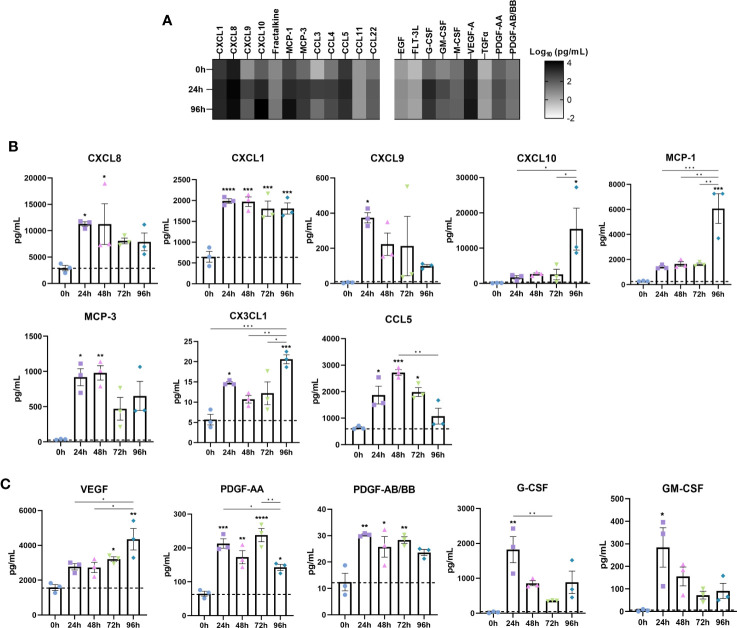
Disease associated upregulation of chemokines is reflected in IBD constructs following induction of an inflammatory response. Chemokine and growth factor content of cell-culture media was quantified by Luminex multiplex platform. **(A)** Heatmap of log_10_ transformed chemokine/growth factor concentrations at 0h, 24h and 96h following LPS stimulation in IBD models. Colour intensity is indicative of the concentration. **(B)** Level of chemokines in 0-96h stimulated IBD models. **(C)** Growth factor levels in 0-96h stimulated IBD models. Data are presented as mean ± SEM from 3 independent experiments; *p< 0.05, **p< 0.01, ***p< 0.001, ****p< 0.0001.

Chemokines and growth factors are important immune regulators in IBD where they potentiate the inflammatory response through binding cognate receptors on nearby cells such as immune and stromal cells. Thus, we measured the temporal release of IBD-associated chemokines and growth factors in the secretome of inflamed IBD models (**Figure 4**).

CXCL8 (IL-8) is a major proinflammatory chemokine released by immune cells, fibroblasts and epithelial cells in IBD (32). Stimulated models elicited a potent CXCL8 response throughout the stimulation period (**Figure 4B**). CXCL1 (GROα), a disease biomarker, was also significantly increased following 24 hours of stimulation, and remained elevated across the stimulation period (33). Although almost absent in controls, CXCL9 secretion was significantly induced upon stimulation, simulating upregulated levels in the IBD mucosa, where it is induced by IFN-γ, and correlates to disease activity in UC (34). Whilst negligible in controls, CXCL10 exhibited a moderate increase during 24-72 hours of stimulation. Of note however, was the substantial increase in CXCL10 observed only at 96 hours recapitulating the significant elevation in disease (35). Mirroring this expression pattern was CCL2 (MCP-1) and IL-6. Downstream angiogenic molecules including VEGF were also upregulated in a time-dependent manner (**Figure 4C**) (36). Similarly, PDGF-AA and PDGF-AB/BB, known to be profibrogenic, were significantly higher in inflamed models than controls, all of which are reflective of IBD tissue/serum (37, 38).

CCL7 (MCP-3), which correlates to the extent of epithelial destruction in IBD patient biopsies, was also significantly upregulated following stimulation (39). Secretion of CX3CL1 (fractalkine) was significantly increased, particularly at 96 hours. Also of note was the increase in G-CSF and GM-CSF, both of which displayed comparable temporal trends. Finally, CCL5 (RANTES), which was found to be abundantly expressed in granulomatous IBD tissue found in CD, was highest following 48-hours of stimulation (40).

### 3.5 Epithelial structure and function is compromised as a result of inflammation in IBD mucosal models

**Figure 5:**
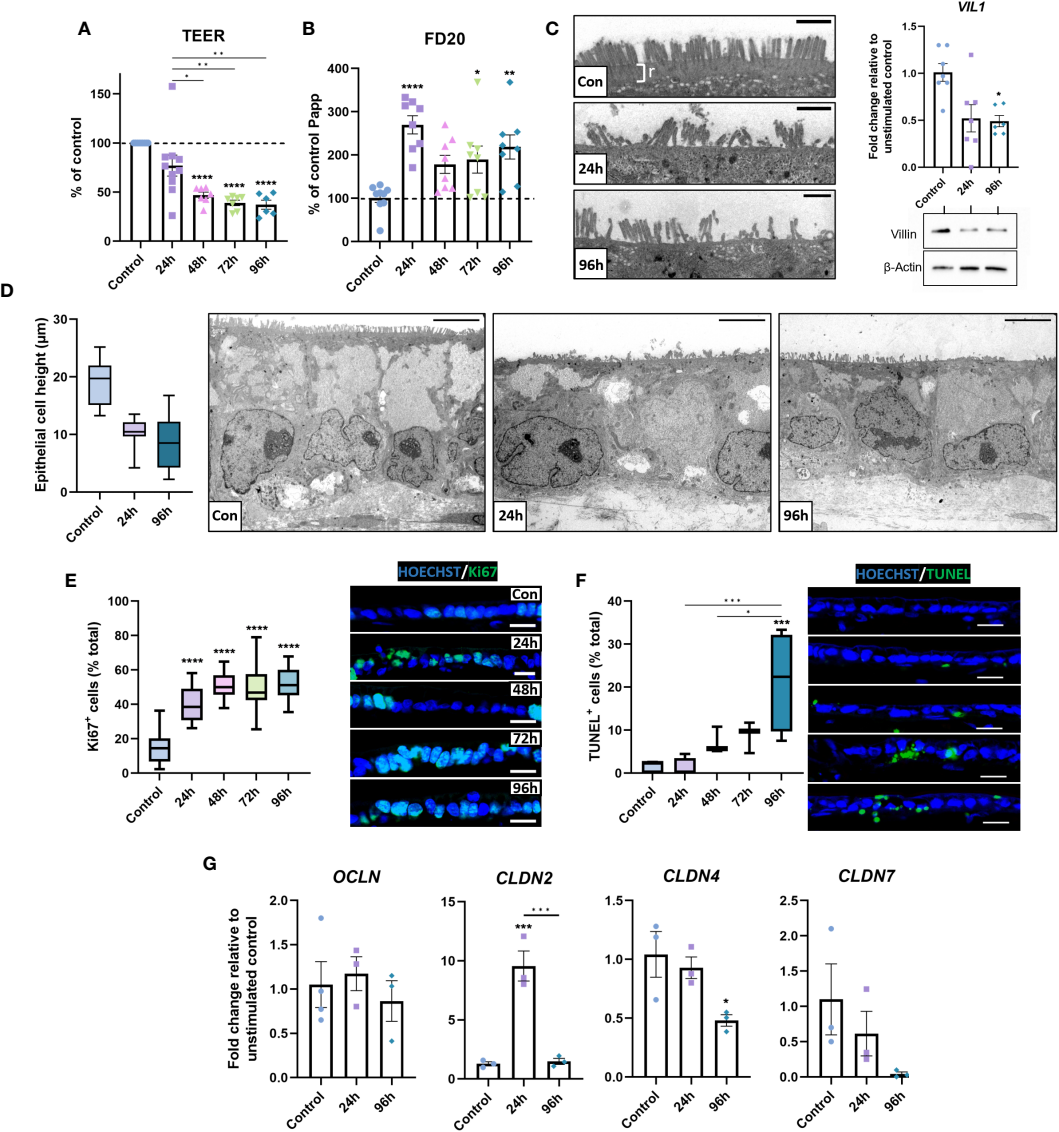
Barrier integrity is compromised in inflamed IBD tissue equivalents. **(A)** TEER was significantly decreased following repeated stimulation with 1 μg/mL LPS. (n = 6-10). **(B)** Paracellular permeability to FD20 was increased in stimulated IBD mucosal constructs. (n = 8-9). **(C)** TEM micrographs of microvilli brush border damage and rootlet (r) loss in stimulated models (*left). VIL1* was quantified by qPCR (n = 6-7) and western blot for villin shown (*right*), β-Actin used as loading control. Scale bars = 1 μm. **(D)** Morphometric quantification of decreased epithelial cell height (*left*) in stimulated models compared to controls. Representative TEM images show loss of cell height and polarisation (right). Scale bars = 5 μm. (n = 26-33). **(E)** Quantification of Ki67-positive epithelial cells as a percentage of total cells (left) indicates hyperproliferative epithelial response in stimulated models. Representative IF images of increased Ki67+ staining (right). Scale bars 20 μm. (n = 11-16). **(F)** Quantification of apoptotic epithelial cells as a percentage of total epithelial cells using TUNEL assay (left) and representative IF images of increased apoptosis (right). Scale bars 20 μm. (n = 6). **(G)** Relative mRNA expression of tight-junction genes OCLN, CLDN2, CLDN4 and CLDN7 in 0, 24 and 96-hour stimulated models. (n = 3-4). Data bars represent mean ± SEM and boxplots represent median and interquartile range. Data is from at least 3 independent experiments, except for TEM data, which was acquired from 2 independent experiments. *p< 0.05, **p< 0.01, ***p< 0.001 and ****p< 0.0001.

Next, we assessed how the inflammatory response affected the epithelial barrier in inflamed IBD models. A significant reduction in TEER occurred following repetitive stimulation, indicating loss of barrier integrity (**Figure 5A**). Assessment of paracellular barrier function using FD20 demonstrated an increase in paracellular permeability (**Figure 5B**), suggesting a leaky-gut phenotype was induced by stimulation.

To elucidate potential mechanisms of epithelial dysfunction, epithelial structure was subsequently investigated. TEM analysis revealed microvilli loss/damage at the epithelial surface of inflamed models, coupled with loss of rootlet visibility (**Figure 5C**). This contrasted the confluent brush border with clear rootlet structure observed in controls, suggesting aberrations to the actin-cytoskeleton at these surface specialisations. To further investigate this, transcript and protein levels of villin were examined, which were significantly depleted in inflamed IBD mucosal models, confirming the loss of an integral cytoskeletal protein that may contribute to epithelial dysfunction. Additionally, the overall epithelial architecture appeared less columnar and polarised in inflamed mucosal models than in controls (**Figure 5D**). Instead, epithelial height was reduced in stimulated models, which exhibited a cuboidal, flattened phenotype.

Areas of pseudostratified epithelium were apparent in inflamed models (**Figure 2A**). To further investigate this, Ki67^+^ quantification was employed to quantify epithelial proliferation. Increased epithelial proliferation was observed in stimulated models (**Figure 5E**). Notably, Ki67^+^ cells were most abundant in pseudostratified areas of the epithelium. TUNEL assays also revealed a time-dependent increase in apoptotic epithelial cells in stimulated models (**Figure 5F**). Together, these data demonstrate increased epithelial turnover in inflamed IBD models, matching observations previously made in UC tissue (41).

Paracellular permeability across the intestinal epithelium is largely regulated by tight junctions and aberrations in tight junction composition are well documented in IBD (42). Herein, differential expression in *CLDN2, CLDN4* and *CLDN7* mRNA levels were observed, but not *OCLN* mRNA in inflamed IBD mucosal models compared to controls (**Figure 5G**). Repetitive stimulation reduced *CLDN4* and *CLDN7* mRNA, which encodes a sealing claudin, and a claudin implicated in regulating paracellular flux, respectively. In contrast, *CLDN2* was increased, which encodes a pore-forming claudin.

### 3.6 Evidence of inflammatory matrix remodelling within the lamina-propria compartment is comparable to fistulising CD

**Figure 6:**
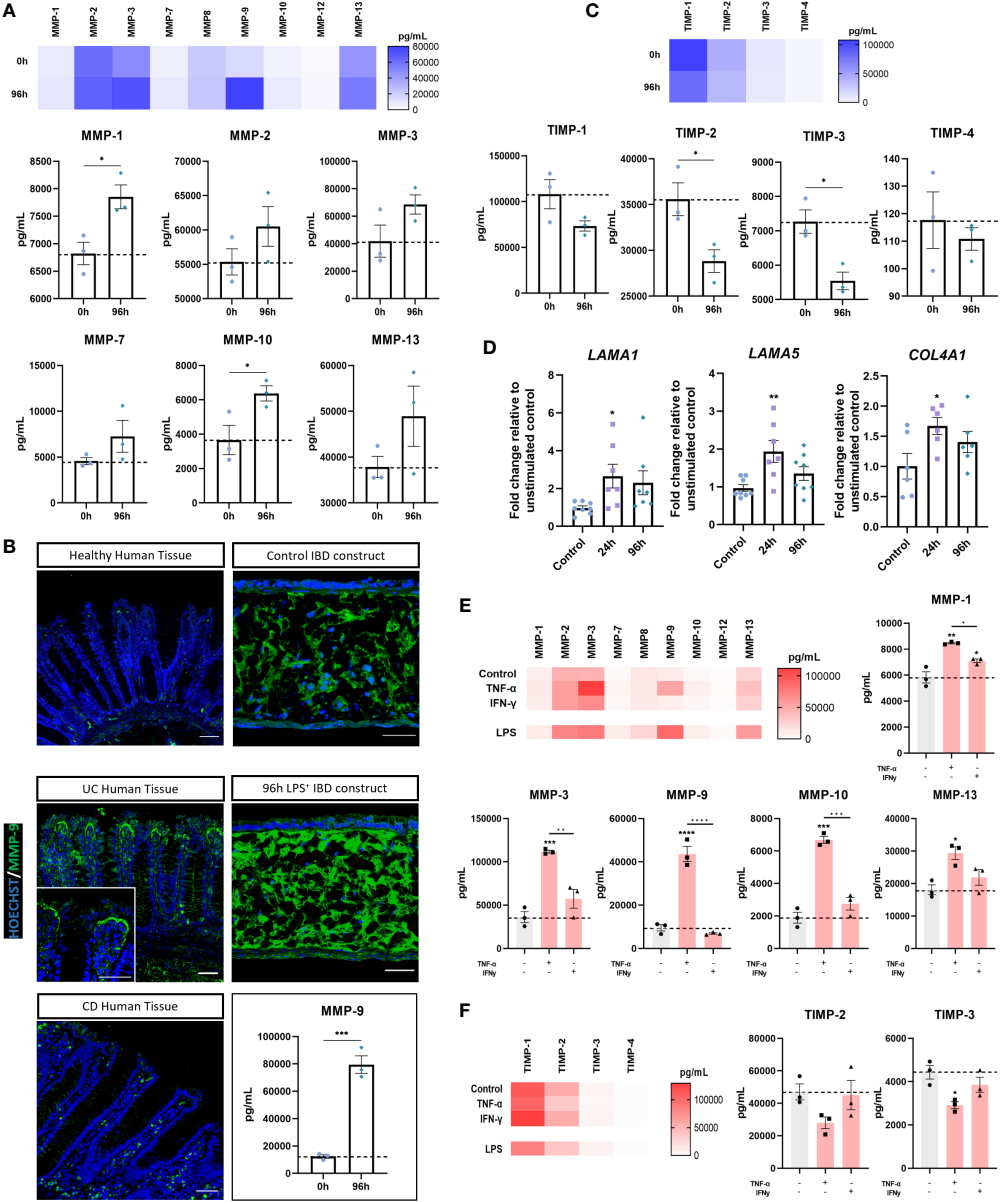
Evidence of stromal reshaping induced by inflammation. **(A)** MMP levels (pg/mL) in supernatant of unstimulated and 96-hour stimulated inflamed IBD mucosal constructs. Heatmap shows relative MMP concentrations *(upper).* Individual concentrations of MMP-1, -2, -3, -7, -10 and -13 are also shown *(lower).* (n = 3). **(B)** IF images of increased MMP-9 in CD and UC compared to healthy human tissue as well as increased MMP-9 in 96-hour inflamed IBD constructs compared to unstimulated controls. Scale bars 50 μm. MMP-9 absolute concentration measured from model supernatants depicts significant upregulation *(lower right)*. (n = 3). **(C)** TIMP concentrations (pg/mL) measured from culture supernatants of control and 96-hour inflamed IBD constructs. Heatmap depicts relative concentrations (*upper*). Individual TIMP concentrations also shown (*lower*). (n = 3). **(D)** RT-qPCR analysis of *LAMA1, LAMA5* and *COL4A1* in control, 24 and 96-hour stimulated IBD models. (n = 7-8). **(E)** MMP release (pg/mL) from IBD models following pro-inflammatory cytokine stimulation. Heatmap shows relative MMP concentration and individual levels of MMP-1, MMP-3, MMP-9, MMP-10 and MMP-13 are also shown. (n = 3). **(F)** Heatmap of TIMP levels in cytokine stimulated IBD models (*left*) and individual levels of TIMP-2 and TIMP-3 (*right*). (n = 3). All data are presented as mean ± SEM from 3 independent experiments for MMP/TIMP studies and 7-8 independent experiments for qPCR. *p< 0.05, **p< 0.01, ***p< 0.001 and ****p< 0.0001.

Chronic inflammation disrupts stromal homeostasis in IBD, generating a proinflammatory mucosal state and extensive ECM remodelling. As such, we next investigated stromal inflammation in IBD models. Inflamed IBD models secreted higher levels of all MMPs quantified than controls (**Figure 6A**). MMP-2, -3, -9, and -13 were the most prevalent MMPs in inflamed models, with MMP-1, -9, and -10 exhibiting significant upregulation following stimulation. Of particular interest, MMP-9 levels were found to be markedly higher than any other MMP in inflamed IBD constructs. (**Figure 6B**). This was of particular importance as MMP-9 is a potential disease biomarker and has been reported to be the most abundant MMP in IBD patient mucosal tissue (3). Immunofluorescent staining confirmed more abundant MMP-9 expression in UC and CD tissue, as well as inflamed IBD constructs compared to healthy tissue and control models. Strikingly, accumulation of MMP-9 was observed subjacent to the epithelial barrier in UC tissues, which to our knowledge is a unique finding of this study. This MMP-9 accumulation was restricted to the upper epithelium and not towards the base of the crypts. Similar observations were made in inflamed IBD models where accumulation was evident in close proximity to the BM.

The TIMP family were also quantified to identify changes in the MMP : TIMP balance (**Figure 6C**). A significant reduction in TIMP-2 and TIMP-3 following stimulation, taken together with an increase in MMPs, confirmed alteration to the stromal niche in inflamed IBD models. *LAMA1* and *LAMA5* upregulation (**Figure 6D**) further indicated remodelling of the extracellular matrix, particularly at the BM. In line with this observation, *COL4A1* mRNA was also increased in inflamed models.

We then sought to investigate the mechanisms underpinning the upregulation of MMPs within inflamed models. To do this, models were stimulated with recombinant cytokines implicated in the pathogenesis of CD that were also significantly upregulated by LPS herein. Both cytokines produced a significant increase in MMP-1, matching concentrations achieved through LPS stimulation (**Figure 6E**). Similar to LPS stimulation, we found that TNF-α, but not IFN‐γ, resulted in highest secretion of MMP-3 and MMP-9 of all MMPs measured. In addition to this, TNF-α but not IFN‐γ, significantly increased production of MMP-10 and MMP-13. TNF-α stimulation elicited higher MMP production across all MMPs quantified than IFN‐γ stimulation, further evidencing the role of TNF- α in mucosal tissue remodelling. Conversely, with the exception of MMP-1, these results indicate IFN-γ alone is insufficient to directly cause MMP-mediated tissue destruction. MMP-3 upregulation is also of clinical importance given its upregulation in CD and UC tissue (43). The MMP-3 concentration observed following TNF-α stimulation exceeded that of LPS stimulation, suggesting TNF-α is strongly implicated in MMP-3 overexpression. Finally, MMP-9 production was considerably increased by TNF-α. However, the MMP-9 response was higher in LPS stimulated models, suggesting multifactorial upregulation.

TNF-α, but not IFN‐γ stimulation also reduced TIMP release in stimulated models (**Figure 6F**). In particular, TNF-α induced a strong reduction in the level of TIMP-2 and TIMP-3, suggestive of increased ECM degradation.

## 4 Discussion

Existing pharmaceutical interventions for inflammatory bowel disease are inadequate as a result of the unreliable and non-translatable pre-clinical models employed in disease research and drug testing. This is especially the case in clinical subsets of IBD such as fistulizing Crohn’s disease, where the scarcity of suitable pre-clinical model systems significantly hinders disease understanding and therapeutic development. Moreover, existing therapeutics mostly target activated leukocytes to curtail the inflammatory response, neglecting other important inflammatory mediators. It is therefore likely that future interventions will target other hallmarks of IBD such as epithelial damage or pathological stromal remodelling – which are often overlooked in the currently available model systems. In this study, we developed a novel 3D *in vitro* IBD mucosal tissue construct that overcomes many of the limitations of existing models and allows the study of immune cell activation, compromised epithelial barrier and stromal remodelling in one model system. This system also provides the unique opportunity to investigate the pathophysiology of fistulising CD.

As a prominent cell type involved in inflammation in the IBD mucosa, macrophages were included in models to recapitulate leukocyte infiltration. Macrophages were co-cultured alongside primary human fibroblasts in Alvetex^®^ Scaffold inserts, creating a tissue-like layer containing an extensive endogenous ECM network. We have previously demonstrated the role of this fibroblast-derived compartment in enhancing the structure and function of the epithelium and the importance of cell-cell contact in epithelial formation (26). In line this with, the overlying epithelial layer in IBD models contains structural, functional and polarisation markers reminiscent of *in vivo* tissue. The instructive lamina propria compartment also supports formation of a collagen IV-rich BM at the epithelial-stromal interface - a mucosal component imperative to epithelial function in health and disease (44).

The inflammatory response induced in models through LPS stimulation manifested key aspects of IBD pathophysiology. For example, histopathological features of IBD were observed including basal lymphocytosis and epithelial defects such as hyperplasia (27, 45). The importance of recapitulating the histopathology of the disease is often overlooked when modelling IBD *in vitro* but is advantageous in assessing histological remission in pre-clinical testing. Inflammatory biomarkers including NF-κB signalling, COX-2 and iNOS were markedly increased in inflamed models, confirming the induction of an inflammatory phenotype. Secretomic analysis revealed the presence of a multitude of cytokines important to IBD pathophysiology and their temporal trends. Increased tissue and circulating levels of many proinflammatory cytokines are well-studied in the context of IBD, with levels of TNF-α, IL-1β, IFN-γ, IL-6, IL-12, and IL-17 as just a few examples increased in this study that are implicated in tissue destruction and disease severity (31). The cytokines were not limited to a particular cytokine signature, namely a CD-typical TH1/TH17 response, or UC-atypical TH2 profile. Instead, secretomic data suggests that stimulated IBD models could be used to represent inflammatory events in both pathologies.

Chemokine expression is also significantly heightened in active IBD tissue compared to healthy tissue and are thus attractive therapeutic targets (46). This chemokine upregulation is also mirrored in inflamed IBD mucosal models. For example, the CXCR8 response elicited by inflamed IBD models mimics the overexpression in IBD which promotes inflammation through signalling pathways including PI3K/Akt, MAPKs and NF-κB (47). Although well studied, downstream effects of this cytokine in IBD remain unclear. Our inflamed IBD mucosal constructs thus allow CXCL8 investigation, a clear advantage over murine models that lack a CXCL8 homolog. Notably, the temporal expression pattern of CXCL10 stimulated our interest as similar trends were noted for CCL2 and IL-6. Unlike other immune mediators, IL-6, CCL2 and CXCL10 were most profoundly upregulated following 96 hours of stimulation, which may suggest pathway association. Whilst no IL-6-CXCL10-CCL2 axis has been identified in the pathophysiology of IBD, one such axis has recently been identified in the SARS-CoV-2 cytokine storm, wherein a correlation between this cytokine axis and severe illness was postulated (48). Further work is therefore required to investigate this within inflamed IBD constructs and IBD. CCL5 (RANTES) was also significantly increased in stimulated models and represents a potential epithelial-leukocyte crosstalk mechanism as has been suggested in IBD tissue (17, 49). Other upregulated inflammatory mediators include angiogenic and mitogenic growth factors VEGF and PDGF, also similar to the disease situation (50). Taken together, the secretomic data obtained from inflamed IBD mucosal constructs are representative of an IBD-like inflammatory phenotype. The importance of having a multicellular, tissue-like system to investigate cytokine and chemokine responses is invaluable since these inflammatory mediators are secreted by immune, epithelial, and stromal cells. However, further work is required to investigate the cell types responsible for upregulation of individual biomarkers within the models.

Epithelial dysfunction and the subsequent ‘leaky-gut’ phenotype is a hallmark of IBD. Despite being well-established in murine models, epithelial permeability has only recently been achieved using *in vitro* models through use of LPS stimulation or recombinant cytokines (13, 14, 51, 52). In line with this, inflamed IBD models exhibited significantly reduced TEER values and increased permeability compared to controls, indicative of barrier disruption. We therefore sought to investigate the cause of this epithelial dysfunction. Striking structural differences were found between control and inflamed IBD mucosal constructs - the latter containing more cuboidal cells that lacked the polarity and epithelial height exhibited by controls. This matches observations in IBD patients and squamous wound-associated epithelial (WAE) cells at areas of epithelial disruption, which are particularly prevalent in fistulising CD during epithelial-to-mesenchymal transition (EMT) (53–56).

Moreover, inflamed mucosal models exhibited clear evidence of microvilli loss/damage and lacked prominent microvilli rootlets. This was confirmed by decreased villin at the transcript and protein levels in inflamed models. Villin is the major F-actin-binding protein located in the cytoskeletal core of microvilli where it regulates cell adhesion and cell death and is thus imperative to epithelial function (57). Loss of villin has been implicated in increased EMT as well as reversible inhibition of brush border assembly and could therefore be implicated in the ultrastructural aberrations observed in inflamed IBD mucosal constructs (58, 59). The lack of visible rootlets may also be a result of reduced actin bundling. These particular findings corroborate those in a recent study in CD patients which reported decreased microvilli height, reduction in microvilli density and rootlets (60). To our knowledge however, ultrastructural analysis of UC patient tissue has not been performed.

Inflamed IBD models also exhibited increased epithelial turnover, which is characteristic of IBD (61). In addition, epithelial pseudostratification and hyperplasia were noted in the inflamed tissues. Inflammation-induced hyperplasia is documented in murine models, UC tissue and UC-derived organoids where it is associated with healing zones (62, 63). Together with the increase in Ki67^+^ cells in inflamed IBD models, we believe a compensatory mechanism may create equivalent ‘healing zones’ within such models. Finally, gene expression analysis suggested changes to tight junction composition that may cause the leaky barrier phenotype observed in inflamed models. In contrast to other studies, we found no significant changes in occludin mRNA expression following stimulation (42). Occludin mRNA is predominantly downregulated in IBD tissues but differential expression has been reported in varying inflammatory states in UC (64). However, inflamed models exhibited a significant increase in *Claudin-*2 which is known to mediate channel formation and the leaky-gut phenotype in IBD. Moreover, decreased *Claudin-4* and *Claudin-7* also match observations in IBD (65, 66).. Claudin-4 is a sealing claudin-family member implicated in chloride reabsorption whereas claudin-7 is a regulator of paracellular flux and epithelial-BM interactions; therefore, depleted levels of either could result in increased barrier permeability. Deletion of claudin-7 has also previously been shown to increase hyperplasia and upregulate COX-2, MMP-3 and MMP-7 in mouse models of IBD, so epithelial-stromal interactions should also be investigated in future work (66–68). Collectively, these data demonstrate that IBD models represent a platform for elucidating IBD epithelial pathophysiology.

Finally, we investigated the applicability of IBD constructs to model stromal inflammatory events. Stromal cells play an active role in the IBD mucosa where they perpetuate chronic inflammation (2). By recreating a lamina propria-like tissue layer containing fibroblasts, we were able to simulate this multicellular contribution to inflammation. Quantification of MMPs and TIMPS confirmed alterations in the stromal niche. MMPs including MMP-1, MMP-9 and MMP-10 were significantly upregulated, mimicking IBD (69). A concomitant decrease in TIMP levels suggests increased ECM-remodelling in inflamed models. Interestingly, the MMP : TIMP expression profile including high MMP-3, MMP-9 and MMP-13 also closely resembles that documented in fistulising CD tissues (9, 70). MMP-9, a biomarker of IBD and mediator of tissue injury, was the most abundant MMP in inflamed models (71). Upon further investigation, we found MMP-9 localisation was similar to that in IBD tissue, particularly UC tissue. Another unique finding of this study was the expression pattern of MMP-9 found in UC tissue, where it was found in abundance at the epithelial-stromal interface. As a type IV collagenase, MMP-9 may result in BM destruction, which may reduce barrier integrity in IBD as well as our IBD mucosal constructs. The intestinal BM is a specialised ECM network implicated in barrier structure and function. *COL4A1, LAMA1* and *LAMA5*, important BM constituents, increased in inflamed IBD models, supplementing very limited existing data on BM remodelling in IBD (72, 73). Models could therefore be used to study basement membrane fragmentation in IBD, particularly in fistulising CD.

We subsequently investigated cytokine mechanisms underpinning MMP upregulation in inflamed IBD mucosal models through addition of recombinant cytokines. Notably, we found that TNF-α, but not IFN‐γ induced significant upregulation of MMP-9, MMP-10 and MMP-13 whereas both cytokines were able to upregulate MMP-1. A correlation between TNF-α and MMP-9 has been outlined in IBD, but as of yet no causal link has been identified (74). Furthermore, TNF-α significantly increased MMP-3 in inflamed IBD models. This is of clinical significance as MMP-3 was recently found to be elevated in non-responders to anti-TNF therapy Infliximab (43).

Collectively, our results successfully demonstrate the ability of inflamed IBD mucosal tissue models to recapitulate multiple pathological hallmarks of IBD simultaneously. Importantly, the model represents an opportunity to study ECM-remodelling events observed in fistulising CD. However, no *in vitro* model truly represents the IBD mucosa, and limitations of these models must be noted. For example, although our tissue construct is more complex compared to existing cultures, there remains further scope to include other cell types that contribute to mucosal health and disease. Another limitation emanates from the cell-lines used. As a result, further work is being performed to include additional cell types as well as replace cell-lines with physiologically relevant alternatives. Nonetheless, the model presented herein represents a valuable research tool that can be used to advance IBD research and pre-clinical drug testing, particularly for drugs targeting macrophage activation, barrier disruption or ECM remodelling processes. Further work is underway to validate a pre-clinical drug testing approach.

## Data availability statement

The raw data supporting the conclusions of this article will be made available by the authors, without undue reservation.

## Ethics statement

Studies involving human tissues were approved by Department of Biosciences, Ethics Committee, Durham University. Human intestinal tissues were collected by Reprocell Europe Ltd (Glasgow, United Kingdom) under appropriate ethical protocols in compliance with local laws and regulations that were reviewed and approved by the West of Scotland Research Ethics Committee (REC Ref: 17/WS/0049). All tissue donors provided informed written consent. Tissues were received at Durham University under a formal MTA agreement and were processed following relevant UK HTA rules and guidelines at the time of publication.

## Author contributions

CM: Conceptualization, Data curation, Formal Analysis, Investigation, Methodology, Visualization, Writing – original draft, Writing – review & editing. ND: Methodology, Writing – review & editing. SP: Funding acquisition, Project administration, Supervision, Writing – review & editing.
