# Supplementary material for: An in vitro model to study immune activation, epithelial disruption and stromal remodelling in inflammatory bowel disease and fistulising Crohn’s disease
Source: Front Immunol. 2024 Feb 12;15:1357690. doi: 10.3389/fimmu.2024.1357690 (PMC10894943; doi:10.3389/fimmu.2024.1357690)
Supplement: Supplementary file 1 [file DataSheet_1.pdf]

Supplemental information

Table 1: Primary antibodies for immunofluorescence and western blotting

Antibody target	Source	Dilution (IF)	Dilution (WB)
Vimentin	Santa Cruz Biotechnology (sc6260)	1:100	
α-SMA	Abcam (ab7817)	1:100	
CD68	Abcam (ab955)	1:100	
Collagen I	Abcam (ab34710)	1:100	
Collagen III	Abcam (ab7778)	1:100	
Collagen IV	Abcam (ab182744)	1:100	
Fibronectin	Abcam (ab32419)	1:100	
E-cadherin	BD Biosciences (610181)	1:100	
Na^+K^+ATPase	Abcam (ab76020)	1:100	
Villin	Abcam (ab130751)	1:100	1:1000
Claudin-1	Abcam (ab15098)	1:100	
ZO1	Abcam (ab59720)	1:100	
CD14	Abcam (ab183322)	1:100	
iNOS	Abcam (ab178945)	1:100	
COX-2	Abcam (ab188183)	1:100	1:1000
Ki67	Abcam (ab118817)	1:100	
MMP-9	Abcam (ab76003)	1:100	
β-actin	Abcam (ab8224)		1:5000

Table 2: Primers for quantitative real-time PCR

Gene	Forward sequence	Reverse sequence	Primer Pair ID
CD68	GTACTGAACCCCAACAAAAC	ATGTAGCTCAGGTAGACCAAC	H_CD68_1
CD86	CCCACTGAATTTTGTGTACC	CTCTAGAGCATAGTAATCACAC	H_CD86_1
VIL1	AAGATGGTAGATGATGGGAG	CAAACGTAGAGCAGGTAATG	H_VIL1_1
CLDN2	CTGAGGAATGACTACTTCCC	ATCTAGAAGACCCTGAATGGC	H_CLDN2_1
CLDN4	GAGCCATATAACTGCTCAAC	AGATAAAGCCAGTCCTGATG	H_CLDN4_1
CLDN7	CATAATTTTCATCGTGGCAG	ATACTCCTTGGAAGAGTTGG	H_CLDN7_1
OCLN	GGACTGGATCAGGGAATATC	ATTCTTTATCCAAACGGGAG	H_OCLN_1
LAMA1	CATACATCACTCATCAATGGC	TAGCGTCTGGTAACAATAGG	H_LAMA1_1
LAMA5	ATCCTATGACTTCATCAGCC	TTGTTATAGAAGAGGGAGAGG	H_LAMA5_1
COL4A1	AAAGGGAGATCAAGGGATAG	TCACCTTTTTCTCCAGGTAG	H_COL4A1_1
